# Remaking time: Cultural semiotic transformations of temporality during the early days of the COVID-19 pandemic and lockdown

**DOI:** 10.1007/s12124-022-09711-6

**Published:** 2022-07-15

**Authors:** Jesse N Ruse, Paul Rhodes, Luca Tateo, Raffaele De Luca Picione

**Affiliations:** 1 Mind and Brain Centre, School of Psychology, University of Sydney, 94 Mallett St, Camperdown,, 2050 Sydney, New South Wales Australia; 2 University of Oslo, Oslo, Norway; 3 Università Telematica Giustino Fortunato, Benevento, Italy

**Keywords:** COVID, Cultural-semiotics, Distress, Time, Coronavirus, Temporality

## Abstract

This paper investigates one aspect of meaning making that occurs in the wake of systemic change. It addresses the question of how time is re-configured by socio-material changes resultant from the COVID-19 pandemic. Employing a semiotic perspective, we aim to describe a process of disruption and distress, which leads to a recognition of the oddness of ‘covid-time.’ This is characterised by distressing ‘suspended waiting’, a despairing frozen temporality. After this, this odd covid-time is semiotically assimilated into the old and familiar. Distressing ‘suspended time’ is transformed into ‘productive time’, ‘normal time’, and ‘transformational time’ as an attempt to regulate affect. By highlighting this semiotic shift, the theory of the Cultural Psychology of Semiotic Dynamics (Valsiner, 2014) is used to highlight how meaning is constructed using cultural resources.

## Introduction

### COVID-19

Beginning in late 2019/early 2020, the SARS-COV2 (Covid-19) virus began to spread from Wuhan, China, to all corners of the world. In early March 2020, the World Health Organisation (World Health Organisation, 2020) declared the spread of the virus a pandemic.

As the outbreak of the virus spread from one country to the next, governments, businesses and citizens began to adopt novel, unexpected and sometimes controversial policies to contain the virus or slow its spread. This included severe restrictions on travel, mandates to stay at home (lockdown; Jacobsen & Jacobsen 2020), social/physical distancing when outside the home (Lewnard & Lo, 2020) and mandatory mask-wearing, among others.

It is in the midst of the calamity of the pandemic that “the loss of a sense of normalcy is widespread” (Walsh, 2020. p.901). The social construction of meaning is foregrounded in times of traumatic rupture to one’s normal experience. Traumatic events can prompt a reconstruction of the meanings in one’s life, such as after bereavement (Gillies & Neimeyer, 2006), illness (De Picione et al., 2017; Freda & Martino, 2015), and organised violence (Park et al., 2012). In the wake of such events, common assumptions concerning the way the world works and our place in it are reconsidered. Brutally, the mind is forced to find meaning making resources to cope with such a cataclysm (Park, 2010; Tedeschi & Calhoun, 2004).

Despite the radical changed brought about by the COVID pandemic, collectively the event is made sense of through familiar and enduring cultural symbolic resources (see also Tateo 2021). The meaning of the event is neither something solely inherent in the event itself, nor completely subjective to the individual, but socially constructed through the resources made available by culture (Valsiner, 2007).

### Time

Since early in the pandemic, scholars have speculated on how time/s might be semiotically reconfigured under the COVID pandemic (Flexer, 2020; Venuleo et al., 2020; Wagner, 2020, Bailey et al., 2020). Time is amenable to culturally-semiotic research if we consider that time is both “real” and something that we make-up to guide our actions. Just as we as humans create, rather than discover, place (Mels, 2016), time is semiotically constituted; mediated by cultural understanding (Adam, 2013; Simão et al., 2015; Tateo & Valsiner, 2015). The experience and understanding of time is highly situated andsocially constructed to properly suit the needs of the context wherein that understanding is grounded.

For example, Nowotny (1994) notes that during the transition from the pre-modern to the modern period, European cultures underwent transformation from “God’s time” to “Traders’ time.” Modern time was conceptualised as linear and could be broken into divisible units of minutes, hours and weeks. Virtues such as ‘punctuality’ and goals such as ‘saving time’ became part of life. The new concept of time fit with in the cultural revolution of the enlightenment era in which progress and transcendence over nature was possible. Later, during the transition from the modern to the postmodern era, time again bends to culture. The laboratory and computer-mediation further widen the gap between humanity and nature, making possible a non-stop reality of availability; a world wherein present time reigns over future time. Such temporal regimes, argues Nowotny, are internalised, changing the phenomenological experience of temporality itself. Such an internalised temporality necessarily constrains the possible ways time can be experienced, by also making possible several ways of talking about one’s experience of time.

Brockmeier (2000) identified several temporal narrative models of autobiographical time found in common cultural discursive artefacts. These include the linear model (narratives told in as a sequence of ordered events occurring one after the other), the cyclical model (narratives experienced as repetitive structures in which the same types of events occur over and over), and the static model (in which narratives are centred around one, often traumatic, event that provides the frozen horizon of meaning for all possible past and future events.) These temporal narrative models gain expression in numerous autobiographical accounts of different types (memoirs, psychotherapy sessions, interviews etc.). Brockmeier contends that these models are culturally reproducible “formatting guidelines” that give an understandable structure to new experiences. These guidelines frame perceptual and affective meaning-making, therefore shaping judgements and actions that follow.

Such guidelines therefore constrain the possible ways in which time can be experienced and expressed. In doing so, they provide members of a culture several possibilities to (unconsciously) choose from when novel experiences are made-sense-of. When a strange and new experience occurs, these formatting guidelines and temporal regimes can be used to make the foreign familiar. The rupture to the ‘normal’ brought about by the COVID pandemic ought to stimulate a search for cultural narratives that ‘make sense’ of the phenomena and regulate affect in doing so.

### Meaning and the Pandemic

The cultural semiotician Lotman (2005) suggests that individual minds are always situated within a larger cultural space. These cultural-semiotic spaces (called semiospheres by Lotman) can be considered an amalgamation and extension of individual minds.

Indeed, these cultural spaces might be regarded as *the v*ery condition of interpersonal communication (Semenenko, 2005). Consider that the perceptual phenomenological experience of each organism is organised by the ecological needs of the specific organism in a specific environment. This means that each person’s phenomenological experience is somewhat unique from all else (these assumptions form the tenets of the field of ecological psychology, see Lobo et al., (2018) for review). Between unique phenomenological realities (or *umwelten;* Von Uexküll & Mackinnon 1926) cultural signs translate messages between persons. Interpersonally, the semiotic universe of signs translates esoteric meanings between persons, while intra-personally this semiotic universe comes to constitute individual experience. It is in this way that the individual is unavoidably using cultural meanings when unconsciously interpreting their own experience.

. Umwelten configurations constitute quasi-stable ecological and symbolic contexts by selecting parts of the environment to be perceived and acted upon. Changes to the environment, therefore, may result in changes in signs and shared meanings in the symbolic universe of meanings. Therefore, cultural signs are constantly changing as new connections are made between new phenomena and pre-existing meanings The individual is themself an active sense-maker, continuously involved with finding culturally meaningful ways of experiencing reality (Salvatore, 2018; Valsiner, 2007; De Picione, 2020).

Valsiner’s Cultural Psychology of Semiotic Dynamics (2007, 2014) contends that each act of interpretation is channelled by generalisable meanings within the cultural milieu. These generalisable meanings comprise the affect-laden ‘universe of sense’ (Salvatore et al., 2019) that each person thinks, feels and acts within. The onslaught of incoming sense data is vitalised through past cultural meanings by foregrounding some aspects of reality while backgrounding others (De Luca Picione & Valsiner, 2017; De Picione 2021), enabling a quasi-stable frame of experience. New stimuli are interpreted by referencing the signs in cultural memory, such that cultural semiotic universe provides the possibility and limits of interpreting new phenomena. The signs within this space include natural language, myth, art and symbols (Valsiner, 2007). As such, phenomenological reality, including the experience of time, is an interpretation based on the internalised cultural semiosphere.

This study explores how changes in the everyday lives of persons in the pandemic might influence temporalities (the subjective experience of time), and therefore the shared meaning of time. The liminal experience of moving from life as it was before the pandemic to an unstable and undefined pandemic-existence prompts the expectancy of a post-pandemic existence. It is this liminal space, between the pre-pandemic and the unknown post-pandemic existence, that time requires definition and understanding.

## Aims

The unique experience of time during the COVID pandemic must be slotted into cultural models that gives it some meaning, otherwise it loses its moorings and becomes impossible to comprehend. As such we expect that the participants of this study will connect cultural narratives with their individual experience via their shaping of time. The focus in this study will be the exploration of people’s use of various cultural narratives to make sense of a novel experience, thereby regulating the affective experience of living through a pandemic.

The aims of the study are twofold: to explore the cultural narratives that are used to make personal meaning of subjective temporality, and to consider how such a process mediates affect.

- This study aims to identify the cultural models of time participants use to make sense of the transition from life as it once was to life under the pandemic. Within the field of cultural semiotics, it is assumed that individuals will use readily available cultural models and metaphors to understand their personal experience of the pandemic.
- This study aims to explore how such semiotic resources regulate affect. In this sense, this study aims to understand how time, used as a semiotic resource, enables participants to cope with the psychological distress of the pandemic.

## Method and participants

### Method and rationale: Diary Study

In order to address the research aims, it was determined that an unstructured qualitative diary method may be employed to capture people’s narrative reflections over the course of the pandemic in their natural setting. The use of diaries to collect research data is an established methodology (Elliott, 1997; Jones, 2000; Lundh, 2015). The unstructured diary in qualitative research is considered “self-revealing record that intentionally or unintentionally yields information regarding the structure, dynamics and functioning of the author’s mental life” (Allport, 1942. p. xii). In non-research settings, personal journals are often used to reflectively and introspectively make meaning of one’s own experience. The use of diaries as a research method is suited to investigating the way that a novel phenomenon, such as the COVID-19 pandemic, is transformed into a culturally meaningful experience.

. Through the process of narration, every act and event necessarily gains a temporal frame (Bruner, 1990) guided by the cultural semiospheres it exists within. A diary study provides an ideal way of exploring how people in lockdown experience time and how their experience of the pandemic is shaped by cultural narratives. Participants were asked to submit diary entries as the pandemic was unfolding in real-time, making it possible to assess the meanings that emerged week-to-week, thereby reducing retrospective distortion. This study was initiated by author three at the University of Oslo (ethics details below). This author asked selected participants to keep a diary during the pandemic for a study of shared meaning-making of disparate individuals in a time of collective crisis.

### Participants

An expression of interest advertisement was made to friends and associates of researchers two and three of this paper. Those invited to participate self-selected to be part of the study. Consequently, there was a bias with regards to those who self-selected to participate. A convenient sample such as this reflects the experiences of predominately white, well-educated, middle-class individuals predominately from Europe and Australia. In order to collect the diaries of individuals in the earliest days of the pandemic, a convenience sample was used to necessarily respond to the urgency of the situation at the cost of a larger or more diverse sample. As such, the results of the study should not be generalised to other populations (e.g. lower-class, materially disadvantaged, non-dominant racial or ethnic groups). There were no formal limitations on who could/could not participate.

Participants were asked to submit one diary entry per week for four weeks and send these via email to the study authors. Single diary entries ranged from a few paragraphs to several pages in length. Participants were told that *“The simple task is to write about your personal experience during the period of emergency. You can write about your feelings, thoughts, actions, using text or also using images. You are requested to send once per week your messages beginning on the week of the 13th of March 2020.”* This start-date was approximately two days after the Italian government imposed a national lockdown in response to COVID-19 (Pepe et al., 2020) and about one and a half weeks before most Australian states imposed severe restrictions for all citizens not engaged essential work (Chow et al., 2020).

All participants were provided with the rational for the study (see Appendix) and gave implicit consent by providing their diaries to author three by email. Ethics approval for the study was granted by the University of Oslo (ref: 585,083). Overall, 29 participants submitted diaries to the study. Participants were instructed to provide one diary entry per week for four weeks, though the number of entries submitted ranged from one to six, with the modal number being four diary entries and the mean number of entries being 3.2.

Overall, given that two of the researchers reside in Australia and two in Italy, participants predominately resided in Australia (17), Italy (9), Denmark (2) and the USA (1). Those diaries written in Italian were translated into English by researcher three before analysis. Overall, 24 participants self-identified as female and five as male. The study authors did not attempt to gather formal quantitative demographic data, given the urgency with which events were unfolding and the use of a convenience sample. That said, qualitative reading of the diaries suggested that most diarists appeared to be well-educated, middle-class professionals, ages ranging from young adults (mid-twenties) to older adults (sixties). No children, adolescents, or seniors took-part in this study. Most participants lived alone or with a partner, and only a very small minority lived with their children or elderly parents.

## Analysis

The method of analysis is largely consistent with a social constructionist paradigm (e.g. Gergen 1985) that argues that persons actively construct their realities by accessing meanings and discourses that are available in a community of speakers. As such, the researchers understand that when the participants write their diaries they are performing ‘acts of meaning’ (Bruner, 1990) which they intend to be understood as significant by the reader.

The qualitative data in the diaries were thematically analysed (Braun & Clarke, 2006, 2012), specifically guided by principles of constructionist epistemology. A brief overview of these theoretical orientations is given below.

- **Thematic analysis informed by a constructionist epistemology**: Thematic analysis involves “thematising meanings” (Holloway & Todres, 2003) that arise in a given text, however these meanings are configured by the participants. An appropriately broad definition of a theme being ‘. . patterned response or meaning within the dataset’ (Braun and Clark, 2006, p. 82). Here, the data are scoured for ‘themes’, ‘meanings’ and ‘discourses’ that participants use to interpret and construct reality, with particular interest paid to the experience of temporality. Epistemologically, these themes and meanings are constructed by the participants and, therefore, could have been constructed differently if social, material, political and cultural contingencies had prevailed.^2^ These themes are then grouped into higher order representations that collectively characterise participants’ lived experience of distress, adjustment and time. The results generated from this step were then interpreted by the researchers through a specific theoretic lens:
- **Interpretative analysis through the lens of cultural semiotics**: This step involved understanding ‘themes’ and ‘meanings’ to be semiotic resources that are culturally constituted. Using a theory-driven approach from the field of the psychology of cultural semiotics (Valsiner, 2014; Salvatore, 2016), the authors explored and interpreted the emergent meanings in terms of semiotic devices, drawn from the larger cultural space, that affect phenomenological experience. Using a theory-driven approach, the researchers aim to make explicit the model of knowledge that is used to make-sense of the data. In doing so, the researchers highlight that their own interpretive and sense-making process (i.e. of data analysis) also occurs within a field and is contingent upon the researchers’ own semiotic universe.

In step a), the first-author researcher read and re-read all diary entries, becoming familiar with the data and searching for ways that participants made meaning of their experience. The first step in analysis involved open coding, wherein all data are coded according to the meanings and themes that the participants used to record their experience. In this step, the researcher coded in a line-by-line manner, “naming segments of data with a label that simultaneously categorizes, summarizes, and accounts for each piece of data” (Charmaz, 2006, p.43). Open coding was undertaken by the first-author and partially coded by the second-author to support trustworthiness. After this, the researcher selected the most significant, salient and frequent codes (focussed coding). These significant codes are then grouped together into over-arching super-themes, which represented an abstracted similarity between the themes subsumed within it (axial coding). After this step, researchers reread all diary entries searching for further instances of the themes that were identified in the previous steps.

The super-ordinate themes generated from this part of the analysis represented both participants’ unique phenomenological experience (Corbin & Strauss, 1990) while also giving insight into how each of the participant’s unique experience draws more generally on culturally available meanings. The interpretive analysis (step b) then involved discussing the themes between the four researchers, wherein the living theory (Whitehead, 1989) of the researchers and their influences in clinical and cultural psychology further informed the interpretation of these themes as semiotic resources that regulate affect in some way.

The researchers aimed to identify how participants operationalised their affective reactions to the transition to life under COVID, under the presumption that this transition would be affectively charged (Pfefferbaum & North, 2020; Qiu et al., 2020). As noted above, this effort to ‘identify’ the way participants operationalised their distress is as much informed by the researchers’ construction of this theme as it was by the participants’ construction. In truth, the data presented below represents a co-construction of the themes as it was informed by both these perspectives.

## Results

The results of this study are organised into analytic themes which represent repeated efforts of multiple diarists to understand the pandemic and cope with the global and local uncertainty resultant from it. These themes represent widely available cultural narratives within this group, given that they were evident in multiple diarist’s entries. The themes detail the ways that multiple diarists made sense of their own personal experience through discourses and narratives that are culturally available to them. These themes are:

1. Distressful psychological disequilibrium:
  - The recognition of non-normality: Participants notice the strangeness of their local and global situation. Such strangeness is accompanied by self-reported distress.
  - Temporalities slow: participants notice that time is not experienced in the linear, forward-moving way that it was before. Time appears to slow or stop as material and social conditions of living through a pandemic are experienced. Time becomes ‘suspended’ while the diarists wait for ‘normal’ life to resume.

2. A semiotic transformation occurs in the way that this new time is experienced. Participants use several familiar cultural narratives to transform ‘suspended time’ to regulate their affect:
  - Return to ‘normal time’: the strange is assimilated into the ‘normal’ by re-coding novel activities (e.g. zoom calls to celebrate St Patrick’s Day) into, in fact, normal activities (socialising, celebrating).
  - A transformation into productive time: the extra-curricular activities that are peppered through the day are semiotically transformed into productive enterprises. Cooking, cleaning, watching webinars, yoga etc. are made valuable by their assimilation into the realm of work.
  - Transformational time: the experience of ‘waiting to return to normal’ is replaced with ‘metamorphosis into something new’. The slowness of suspended temporality is individually experienced as a personal reflective space, and collectively as a necessary global re-prioritization of values.

### Distress as experienced by the participants

- situating shattered temporalities in the time of COVID - the recognition of non-normality

One of the more evident initial consequences of the pandemic was the sudden change to everyday routines of work and socialising. The diarists describe becoming estranged from normality either through lockdown (i.e. mandated social distancing in their home) or by strangely reduced contact with others (transitioning exclusively to ‘Zoom meetings’, keeping distance from others in public, etc.):

- *It took me years to learn to be alone, but never like now I realize that I need human contact. I need to hear from a friend and say “let’s drink it’s been a hard week I need alcohol” or the “come on let’s go out tonight”. –* Female, Italy, week 1.
- *I felt really lonely during the first few days. I tried to make the house lively by playing music loudly and keeping all light on. And when I was out for walking, it seemed that everyone wanted to keep away from others, which is totally right to protect people from the virus, but still, I miss days that people can smile and say hello to strangers.* – Male, Denmark, week 1.

Prominent characteristics of these changes are reduced physical contact with others, changed routines of work and socialising, and a keen awareness of the disruptions to local, national and global life. Not all participants explicitly express that these changes initially caused distress. Indeed, some participants express some satisfaction (at least, initially) that they can cut out daily commutes or spend more time with their children at home.

b. stretched temporalities and time: living in suspended time - ‘Dead time’, ‘wasted time’

As the distressing effects of the pandemic started to amass during the first weeks of lockdown, diarists increasingly began to note a new and perhaps unexpected source of distress: time was no longer flowing the way it normally had. Diarists struggled to find an appropriate way to make sense of temporality whilst within the liminal ‘in-between’ period of pre- and post-covid existence. Many of the participants express that their time, once so meaningful and productive, starts to become “wasted” and “dead.”

- *I am simply wasting my time waiting that all this will be over. I am fluctuating into time. Not really using it, not really wasting it, but not able to make it better.* – Female, Italy, week 4.
- *I can’t handle it. And so I live by two hours in two hours, no longer. Waiting for a time that doesn’t pass and I’m afraid of. –* Female, Italy, week 1.
- *This week I reckon I have lost cognition of the time passing by. Yesterday I thought it was Tuesday: nope! Thursday. I don’t know if this is good or bad. Thing is I feel like the time is passing more slowly than before.–* Female, Italy, week 2.
- “*Sometimes it feels very hard to be “human” in the isolation period, not to mention being productive” –* Male, Denmark, week 2.

Diarists noted how they found it difficult to make plans and extend their lives into the future. Instead, they are simply stuck ‘waiting’. In the liminal space between life as it normally was and life as it will be after COVID, the participants are forced to relinquish future-planning and live in a suspended ‘now’. The only time that matters is the present and how to deal with what one can do on a day-to-day basis whilst the world sorts itself out.

Phenomenologically, the diarists characterise suspended time by an inability to plan, a lack of social and work activity, and a general angst which does not allow for such idle time to be restful. Generally, the participants are unable to live satisfactorily in this suspended time as they complain of not feeling “human” within it and unable to live forwards through it. The sudden arrival of suspended time prompts a way of understanding and making meaning of it. The following are examples of attempts the diarists make to restore meaning to time:

### semiotic resources to regulate distress

The seemingly undesirable experience of living suspended time prompts an effortful meaning construction process by searching the semiotic universe for answers. The Semiotic Cultural Psychosocial Theory posits that the mediating role of cultural representations gives meaning to one’s experience, thereby altering it (Valsiner, 2007). The affective dimension of this theory further suggests that affect can be regulated through successful use of particular representations (Salvatore & Freda, 2011) that are available in a particular semiotic universe (Lotman, 2005). Below we document the representations/meaning that participants use to achieve affective regulation.

- returning to “normal” time

One way that participants attempt to escape suspended waiting time is by re-engaging in culturally significant activities that connect isolated individuals with others. In this way, the lost temporality marked by ‘waiting’ is returned to ‘normal’ by re-connecting the personal and the collective through culturally universal activities. For example, participants were able to achieve this through connecting with culturally significant event such as Easter and St Patricks day:

- *Then on March 17th with a group of friends scattered around the world we decided to celebrate St. Patrick via Skype and it was a wave of positive energy that accompanied me all week. –* Female, Italy, week 2.
- *On St. Patrick’s evening, the first time we celebrate it at home and that Skype call that brought me back in the world, for smiles, for laughter, the mess and the fact of being there, we were there together, far, but close Like never before –* Female, Italy, week 2.

The restoration of the meaningfulness of time is achieved by the return of the grounded events that populate our calendars and give social life meaning. The excerpts above reference one such cultural event (St Patrick’s day) which effectively brings isolated individual into the social fold. However, many such events occurred over the course of our data collection including larger events (e.g. Easter) and smaller events (e.g. daily coronavirus update made by the government/media).

b. The management of suspended time – affect regulation through ‘productive time’ and ‘transformational time’

Whilst dwelling in suspended time, the diarists become anxious, despairing, and frustrated. Their efforts are therefore directed towards regulating their states via activities that are not only, in and of themselves, enjoyable, but also qualitatively change the nature of time itself. The participants attempt to connect their experience with a cultural meaning that is considered worthy, legitimate and commendable: productivity. Indeed, the participants imply that for time to be meaningful, it must be productive in some way. This includes cooking and cleaning, exercise, yoga, attending webinars, calling friends on Zoom etc.

- *I am trying to cope with this forced quarantine doing my best: webinairs, cooking, cleaning, decluttering, calling people I know are alone in their houses… I try not be too exposed to the news or my panic will devour me.* – Female, Italy, week 3.
- *I rejoice in modern technology; embrace online yoga classes, live streamed gigs and trivia, speaking with friends and family more, playing games with my friend’s children online. There’s time for cooking, art, reading, thinking, watching, listening, quiet; just so much more time. –* Female, Australian, week 3.
- *Everyday I exercise. Everyday I call someone or I keep myself busy with a project: a baking project, a reading project, a dubbing project (I am a dubber as a hobby)…* – Female, Italy, week 3.

In these examples, we see our participants attempt to neutralise the threat of suspended time by imposing a familiar order upon it. This is primarily via personally meaningful and collectively legitimated activities that restore a sense of productivity and structure. Time is therefore restored from ‘suspended’ into ‘personally meaningful’, mediated by activities that are culturally significant as being productive. These include those listed in the quotations above, but also include the ‘smart working’ activities of those who are either forced to work from home or adjust to new office life. What we might otherwise call ‘leisure time’ (if it were not for the pandemic unfolding in the background) is being made to increasingly resemble work-time. This is achieved by attempts to mirror the structure of work-time (the importance of creating a ‘routine’), needing to upgrade one’s skills (baking, webinars, home work-outs), making ‘appointments’ for calls to friends or online classes, coping with ongoing delays and adjustments (attempting to ‘work around’ the pandemic), the need for time to be ‘used’ in the pursuit of some ends (e.g. having a ‘pandemic project’), and the overt appreciation of having work to do.

c. ‘Waiting time’ becomes ‘transformational time’:

Another way diarists attempt to disavow the uncertainty generated by the pandemic is by attempting to transform suspended time into ‘transformational time’. Transformational time may be defined as the gestational, incubational period that precedes social or personal metamorphosis. In this narrative, a new world is created through the necessary destruction of the old world. The pandemic acts to correct the course of society which hitherto has been sailing in the wrong direction. The diarists make sense of their waiting as if it is a necessary, though uncomfortable, event which ought to remind each of us individually not to take the world for granted. The affective consequence of such a semiotic transformation is that the previously nervous and uncomfortable ‘waiting time’ begins to feel hopeful and catalytic.

- *I had come to think that the Planet needed to breathe: we were acting like we were its virus. High temperatures, forests burning like our lungs are burning now … Nature has its way to make us listen to her. Or maybe this had to happen, no other hidden meaning. I’d like to think of this as a lesson to learn: take back our time, stop running, listen to ourselves, take care of our loved ones. We were so loud that we couldn’t listen to this scream, and now we MUST stop.-* Female, Italy, week 2.
- *I start to feel more like it is possible that we’ll come out the other side of this somehow and establish a new normal. There will always be both tragic and wonderful things about change. I try to remind myself about this on a daily basis. I plan to write again next week.* – Female, Australia, week 4.
- *All we can do is to take care of the “here and now”. So maybe the lesson is: life is really ‘here and now’, keep it simple. Do the things we love to do, stay with the people we love, care about the people we love, give time a value.* – Female, Italy, week 2.

Diarists make reference to the potential for the event to be transformational in a collective sense (i.e. to change social and institutional arrangements) and in an individual sense (e.g. as a reminder to one’s self not to take for granted time with loved ones etc.). Such a semiotic move allows participants to experience time not as suspended (e.g. ‘waiting in vain’), but as a necessary recovery that will restore health to the world and to the individual.

## Discussion

The results here indicate that the participants are immediately affected by the strange conditions brought about by the COVID pandemic. In many cases, contact with others is suddenly removed or drastically changed by the pandemic. Early clinical research, predominately measured by questionnaire and other quantitative methods, suggest that the anxiety-inducing effect of the pandemic was moderated by persons felt sense of alienation (Zhu et al., 2021) and loneliness (Robb et al., 2020). Not only were the diarists dealing with the distress generated by the consequences of the pandemic, but there were fewer people physically around to provide validation and comfort when they are feeling distressed.

The diarists are quick to point out that they feel immediately affected by a feeling of non-normality from the first days of the pandemic. The theory of Cultural psychology of Semiotic Dynamics suggests that affect and perception go hand-in-hand (Branco & Valsiner, 2010a, b)Tateo, 2019). The environment exerts an influence on an individual such that it affects him/her, and such affect foregrounds or backgrounds certain elements of perception that thereby give the individual his/her perceived reality (Salvatore et al., 2019). These diaries reveal the immediate affective force of the pandemic and how this thereby shapes diarists’ perception of time. In this case, aspects of temporality are suddenly foregrounded during a distressing pandemic.

Such a finding highlights the social dimension of time. Henri Hubert, in his study on religious life and its assimilation into the social (1904), suggests that collective life segments time *qualitatively*. Time is tied intimately to social and collective life, such that it is the rhythms of life that give each hour, day, week etc., it’s qualitative feeling (see also Lefebvre 2004). Hubert contends that it is the ‘things’ of social life, the objective activities with all their intensities and particularities, that give segments of time qualitative feelings (see Munn 1992, p.52 for brief overview of Hubert’s anthropology). For the diarists of this study, the rhythms of social and collective life are disturbed by the infection control measures. It is therefore no surprise that the participants describe a phenomena where the passage of time no longer ‘feels’ the same.

For many in the study, this initial temporal change is characterised as ‘suspended time’, where the clock ticks over from one hour to the next, though there is little feeling that time is moving forward. For the middle-class working participants in this study time is most ardently fixed to activities that are “productive”. ‘Suspended time’ is felt most strikingly in contrast to feelings of productivity and social activity, the cultural forces that often imbue time with significance. Nowotny (1994) suggests that in the period of late modernity that we now find ourselves, time is experienced in budgetary terms, such that it is something to be used, consumed, and exploited. Zerubavel (1987) points out that our culture has many ways of symbolically assigning importance to durations that are spent productively. For instance, expressions (in English) suggest that one can “spend” their time well or “waste” it by being idle, and points to modern “schedule” as an obsession with the correct use of every particle of time (Zerubavel, 1985).

Suspending normal time causes a reaction in the opposite direction. We see as an effort to restore some sense of normality by connecting the experience of lockdown with the culturally admirable duty to be busy, working, and always improving. As such, through engaging in otherwise banal everyday activities such as cleaning or watching webinars, the participants’ experience of time passed through the filter of the cultural sign ‘productivity’. Such a semiotic transformation has the affective consequence of regulating distress and is used (either intentionally or unconsciously) to return a feeling of normality. In this sense, by re-establishing the normality of full daily routines, the diarists can heal the semiotic rupture brought about by ‘suspended time’.

This effort at the construction of ‘productive time’ links personal tasks with the culturally prized value of industriousness. The historical roots of such might be linked back to the industrial ethics of Calvinsim and Taylorism, and cultural ideologies such as the protestant work ethic (Mudrack, 1999) and newer forms of neo-liberal capitalism (Sugarman & Thrift, 2020). The long history that links feelings of worthiness to time management routines and the mandate to ‘keep busy’ is beyond the scope of this paper, but can be found elsewhere (Sugarman & Thrift, 2020). What is notable from the data in this study is that time is conceived as something that can be spent in the purchase of achievement, self-growth, and creativity.

It is notable that affect is managed through demarcating time into separable units that take their form from the duration of certain activities. The seemingly unending time of the outside world continues indefinitely (when can we return to work? When will the vaccine be available? When will the curve flatten?). Nevertheless, it is possible to regain personal control of time by creating micro-temporalities that structure the days, weeks and months. These temporalities are created by populating time with activities that effectively create separatable units (the time of the activity itself, the time between activities, the regularity with which an activity needs to be done). As such, new temporalities are created that have divisible units, thereby creating an affectively pleasant quality of experience.

It must be noted, at this point, that the vast majority of the pariticpants in this study were female and we cannot ignore the role that gender plays in influencing how time is experienced in family and individual life. Davies (1989) described feminine time as being more “relationally oriented”, partly due differences in child-rearing time commitments and available career options (e.g. higher representation in nursing, teaching, lower in construction and agriculture). This refers to the relatively greater focus that women are expected to have in ensuring that relationships are nurtured and maintained. Predominately, women also tend to be the keepers of family schedules and exert more control over the organisation of time in the household (Daly, 2002; Deem, 1996). The combination of a relational orientation to time, mixed with the responsibility to take on extra domestic duties can lead to womens time being less linear and more cyclical.

The diaries point to some differences in the domestic schedules and social routines of the participants and their experience of disrupted time. Historically, gendered dvisisions in the labour market and in the household have meant that representations of the ideal female identity is suited to acquiesing the needs of others (Odih, 1998, 1999). The women in this study would often regard the loss of embodied relational time (i.e. time physically spent with others) as one to the most stark consequences of their experience of suspended time. As such the loss of embodied relational time came to be a characteristic of suspended time in the highly gendered cohort participating in this study.

Whilst ‘feminine time’ is structured (relative to ‘masculine time’) more around the duties of caregiving than bread-winning (Daly, 2002), both genders live under the constraints of modern capitalism. The time discipline of capitalism is imposed on all equally (and therefore unfairly), and the demands to be productive are felt by all (Bryson, 2007). There are some differences in the way that professional productivity (paid work) and household productivity (unpaid work) transform time.

The majority female diarists in this study place an emphasis on time becoming productive when it is used to connect with others. Women perform greater amounts of invisible, affective connective labour (Boler et al., 2014), which involves tending to the social bonds that enable individuals to work harmoniously together. The semiotic transformation into ‘productive time’ is therefore particularly influenced by gendered cultural narratives, as well as by wider cultural narratives around industriousness.

Another temporal semiotic transformation involved understanding suspended time as ‘transformational time’. ‘Transformational time’ is long and arduous, and it does not have the industrious character of ‘productive time’. Instead, it is an opportunity to make a lesson of the situation; to make sense of the senseless experience by seeing it as an opportunity to learn or transform in some way. Indeed, in his holocaust memoir and treatise on coping with trauma, Viktor Frankl artfully describes how suffering (in his case, in a prisoner of war camp) is much more difficult to bear when such suffering is deemed meaningless (Frankl, 1985). Similarly, the painful and uncomfortable experience of the pandemic is experienced as the painful growth that follows a trauma. For the individual, this involves ‘slowing down’ and taking time attending to the ‘important things.’ Similarly, for the collective, it becomes Noah’s-ark-type re-boot of society, which was somehow always needed, but only just arrived at.

In another study aimed at exploring meaning-making during the pandemic, Venuleo et al., (2020) found that persons often made sense of the pandemic as a prompt to ‘reconsider social priorities’ and ‘reconsider personal priorities’. They found that persons often understood the pandemic as a ‘turning point’; a generative event that, by its consequences, has made us see the folly of our pre-covid existence. The event is marked as the point when the world could no longer sustain the damage being done to it and forced us to stand-back and return to proper living. This idyllic ‘proper living’ involves spending more time with family, treating the earth more kindly, and ‘slowing down’.

Indeed, it may be that it is because of such collective transformation that an individual transformation becomes possible. The transformation of suspended time in the collective sense prompts a need to reconsider the currency given to one’s own personal time. Indeed, popular idioms in health messaging such as “We are all in this together” and “Stay at home. Protect the NHS. Save Lives” aim to emphasise the inseparable nature of the individual and the collective. The damage that the pandemic inflicts upon macro structures such as the economy and the NHS are composite effects of multiple micro effects on individuals. Similarly, it is through micro-revolutions in priorities such as ‘slowing down’ and ‘caring for those around us’ that the world will be able to heal from the pandemic. In this sense, psychological transformations such as slowing down and giving time more “value” are the result of real, material, systemic changes in the way that social bonds are created and maintained.

It must be re-iterated that this sample is largely homogenous with regards to gender, social class and education and any generalisation beyond such a group must be done with caution. A limitation of the study is that there was no formal demographics data obtained which specifies the age of the participants. As such, despite age being an important moderator of meaning-making, no such comment here can be made regarding such. The study is also somewhat limited by the narrow class range (middle class) of the participants, as well as higher representation of women. However, the study aims not at generalisability nor representativeness, but aims to understand how diarists made sense of a novel experience through the use of cultural signs familiar to them. While a different population may have yielded different insight, contingent on the different cultural spaces they inhabit, a population such as this give insight.

Time in this sense is qualitatively transformed by connecting the unusual experience of the pandemic with other unusual experiences (such as traumatic personal injuries, justifiable wars) which are necessary but uncomfortable, and require anxious waiting in order for recovery to take place. This allows for the slowness experienced in time to be understandable and calm. It is in this sense that it allows for affect management.

## Conclusions

The guiding theme of the analysis was the experience of time. Culture connects the unknown to the known; the world of personal experience with the world of meaning. While each diarist experiences temporality in a unique way, such experience must pass through the mediating layer of culture to gain meaning, thereby gaining a collective quality through its communicability. This analysis showed what diarists made sense of the foreign phenomena of suspended time through commonly available cultural narratives.

The analysis begins by explicating the ways that participants experience and construct their distressing “shattered” temporality. The diarists wrote of a disruption to the taken-for-granted notion of time flowing normally from one hour, day and week to the next. The social ‘things’ that make up life (such as work, socialising, planning etc.) are interrupted and a keen awareness of such a disruption sets-in. Diarists then find themselves in a liminal zone between pre- and post-covid existence. The automatic sensemaking processes are no longer able to regulate inter- and intra-subjective domains of experience. Affectively, time takes on a different quality. Temporalities slow or stop, and feelings of strangeness, fear, anxiety, loneliness, and disquiet are amplified. Time is ‘suspended’; diarists complain of waiting for a future that might not come.

This is followed by diarists’ attempts to reconstruct normality through management of temporalities. To neutralise the threat of suspended time, new ways of making meaning of the experience of the pandemic are discovered. What is interesting is that the affective management of temporalities occurs through the recruitment of semiotic resources that are contingent upon the cultural semiospheres that participants exist within. Temporality is rescued from strangeness by returning to the normalness of pre-pandemic times. What is novel about such an affective regulation strategy is that it is guided by temporal ‘formatting guidelines’ or cultural codes that existed in pre-covid cultural awareness. The new application and reformulation of these cultural codes in turn changes their cultural meaning while re-inscribing them into the semiosphere.

The diarists used the following cultural codes to make sense of their pandemic time. Firstly, in a reaction to the suspension of normality, diarists attempt to re-normalise their lives by returning to pre-covid celebrations. An attempt to assert upon time that it is no different now than before; that the essence of our sociality has not changed. Secondly, there are efforts to make pandemic time feel useful and productive. Specific activities and structures are created that resemble work-time. The allure of being productive, upgrading one’s skills and creating ‘projects’ to fill one’s time promises to offer relief to suspended time. Lastly, the slowed temporarily is made-sense-of as a meditative, reflective time. It is a liminal re-organising period which, while uncomfortable, is necessary to metamorphosis into a new personal and collective reality.

The study was able to show that diarists were able to make sense of their experience by integrating their unique phenomenological experience of time into the already-established semiotic universe of meaning. The experience of time is made subjectively meaningful by the interactive use of cultural signs that characterise the experience of time *as* meaningful. What is more, the meaning of the experience of time modulates its affective feel. While slowed time spent anxiously ‘waiting’ is distressing, slow reflective or transformative time is palatable. The global and local contexts furnish the environment with possible meanings and the individual has at her disposal the propensity to use these meanings to regulate affect. To this end, we have shown here that cultural devices exist that are able to constitute the affective reactions to the pandemic.

## Data Availability

The datasets generated during and/or analysed during the current study are available from the corresponding author on reasonable request.

## Appendix 1: *The advertisement for participation in the current study made and disseminated by researcher three*:

“I am a professor at the University of Oslo. This is a study about the experience of people in a time of anxiety and preoccupation.

People are facing a moment of concern and an exceptional experience, due to the spreading of the COVID-19 epidemy. Some people are affected, some other are in quarantine. Some people in different countries are simply requested to stay home.

I ask whoever is willing to share his/her experience of these days to join a qualitative international diary-study.

The simple task is to write about your personal experience during the period of emergency. You can write about your feelings, thoughts, actions, using text or also using images. You are requested to send once per week your messages.

I will collect your diaries and analyse them in order to understand what we share as human beings facing such difficulties. All your diaries will be treated anonymously and no personal information except your age, gender, town and job will be retained or disseminated. You can withdraw at any time from the study. Only a summary of results will be published, without individual information. At the end of the study, I will tell you about the findings.

If you want to participate and you give your consent to the terms above, just post here writing accept, and I will include you in a private group where you can write your posts for a period of 1 month. You can also invite other persons who can be interested in participating.

## Informazioni sullo studio

Sono un professore dell’Università di Oslo. Questo è uno studio sull’esperienza delle persone in un momento di incertezza e ansia.

Le persone stanno affrontando un momento di preoccupazione e un’esperienza eccezionale, a causa della diffusione dell’epidemia di COVID-19. Alcune persone ne sono colpite, altre sono in quarantena. Ad alcune persone in diversi paesi viene semplicemente richiesto di rimanere a casa.

Chiedo a chiunque sia disposto a condividere la propria esperienza di questi giorni di partecipare a uno studio-diario internazionale qualitativo.

Il semplice compito è scrivere sulla tua esperienza personale durante il periodo di emergenza. Puoi scrivere dei tuoi sentimenti, pensieri, azioni, usando il testo o anche usando le immagini. Si richiede di inviare i messaggi una volta alla settimana.

Raccoglierò i tuoi diari e li analizzerò per capire cosa condividiamo come esseri umani che affrontano tali difficoltà. Tutti i tuoi diari saranno trattati in modo anonimo e nessuna informazione personale esclude la tua età, sesso, città e lavoro sarà conservata o divulgata. Puoi ritirarti in qualsiasi momento dallo studio. Verrà pubblicato solo un riepilogo dei risultati, senza informazioni individuali. Alla fine dello studio, ti parlerò dei risultati.

Se vuoi partecipare e dai il tuo consenso ai termini di cui sopra, pubblica semplicemente qui scrivendo accetta e ti includerò in un gruppo privato in cui puoi scrivere i tuoi messaggi per un period”.

## Footnotes

2 That said, this analysis requires us to acknowledge a social-constructionist epistemology with consideration to both participants’ constructions of their distress, and the researchers’ constructions of ‘themes’. On this latter point, we much acknowledge that themes are not passively found in data, but that they are identified and configured somewhat by the researchers’ interests (Ely et al., 1997).
